# Controversies and Perspectives in the Current Management of Patients with Locally Advanced Rectal Cancer—A Systematic Review

**DOI:** 10.3390/life15071011

**Published:** 2025-06-25

**Authors:** Roxana Elena Stefan, Rodica Daniela Birla, Mircea Gheorghe, Daniela Elena Dinu, Petre Angel Hoara, Diana Ciuc, Valeriu-Gabi Dinca, Silviu Constantinoiu

**Affiliations:** 1 Department of General Surgery, Carol Davila University, 020021 Bucharest, Romania; roxana-elena.stefan@drd.umfcd.ro (R.E.S.); daniela.dinu@umfcd.ro (D.E.D.); petre.hoara@umfcd.ro (P.A.H.); silviu.constantinoiu@umfcd.ro (S.C.); 2 Faculty of Medicine, Titu Maiorescu University Bucharest, 031593 Bucharest, Romania; diana_ciuc@yahoo.com (D.C.); dincagaby@yahoo.com (V.-G.D.)

**Keywords:** locally advanced rectal cancer, total neoadjuvant therapy, complete response, non-operative management

## Abstract

Traditionally, the therapeutic approach to rectal cancer has involved neoadjuvant chemoradiotherapy followed by surgical resection, and, in some cases, adjuvant chemotherapy. This study aims to present current advances and ongoing controversies in the management of patients with locally advanced rectal cancer (LARC), with a particular focus on clarifying the role of total neoadjuvant therapy (TNT) in contemporary treatment strategies. Methods: We conducted a systematic literature review in Medline/PubMed using various keyword combinations, including “rectal cancer/neoplasia” and“therapy” or “neoadjuvant therapy” or “TNT”, and included articles published between 2015 and 2025. Results: The association of neoadjuvant radiochemotherapy with preoperative systemic chemotherapy has led to the current concept of total neoadjuvant therapy. The advantages of preoperative chemotherapy include better patient compliance, a decrease in the rate of local recurrence and distant metastases via the early destruction of infra-clinical micrometastases, and higher rates of pathological complete response. All of these have led to the inclusion of this strategy in treatment guidelines for patients with locally advanced rectal cancer. Conclusions: However, the selection of patients with advanced rectal tumors for optimal therapy requires comprehensive imaging assessments, molecular and genetic testing, and a multidisciplinary team to determine the most appropriate total neoadjuvant therapy approach.

## 1. Introduction

In Western countries, rectal carcinoma (RC) is one of the leading causes of mortality. According to Globocan 2022, RC ranks as the 10th most lethal type of cancer, with 343,817 cases, accounting for 3.1% of all cancer-related deaths [1]. Its incidence is steadily increasing, particularly among younger populations [2].

RC differs significantly from colon cancer, with which it is often grouped, both in clinical and biological aspects and in terms of management, therapeutic approach, and surgical intervention [3,4].

Locally advanced rectal cancer (LARC) is defined as stage II (T3 or T4) or stage III, where lymph node metastases are present [5], accounting for 80% of newly diagnosed RC [6].

The objectives of advanced RC treatment are to prevent locoregional recurrence (LR) and distant metastases while maintaining patients’ quality of life. Surgical treatment is one of the key pillars of the therapeutic strategy for RC patients, alongside radiotherapy (RT) and chemotherapy (CT).

Over time, several complementary therapies have been used for RC, with RT being the most frequent. The most commonly used regimens for RC irradiation include long-course radiotherapy (LCRT) and short-course hypofractionated radiotherapy (S-RT) [7].

LCRT consists of conventional fractionation with doses of 1.8–2 Gy per fraction, administered in 25 to 28 daily fractions over five days per week, reaching a total dose of 45–50 Gy. Concurrently, radiosensitizing CT is administered as chemoradiotherapy (CRT). Initially, 5-Fluorouracil (5FU) was used at a dose of 1200 mg/m^2^ per day, later replaced by Capecitabine at 825 mg/m^2^ twice daily, which is currently the most frequently used regimen. Surgery is typically performed 4 to 12 weeks after the completion of radiotherapy, with the most common interval being between 6 and 8 weeks [8].

S-RT involves doses of 5 Gy per fraction administered for five consecutive days over a week, with a total dose of 25 Gy, without concurrent radiosensitizing CT. Surgery is performed within 10 days after RT or delayed, similar to the LCRT approach [9,10].

For over 20 years, various adjuvant and neoadjuvant therapeutic strategies have been developed, significantly improving prognosis alongside appropriate patient selection and standardized treatment methods [11,12].

The objectives of neoadjuvant therapy include reducing tumor stage until complete tumor cell eradication, achieving a complete clinical response (cCR) to enable a wait-and-watch (WW) strategy, and preventing local recurrence (LR) after surgical resection.

Although the standard treatment for LARC consists of neoadjuvant CRT followed by surgery and adjuvant CT, there is a current shift toward neoadjuvant CT in the form of total neoadjuvant therapy (TNT). This approach appears promising in improving pathological response, LR, metastases, and potentially OS [13,14], allowing the selection of patients with advanced tumors for non-operative management (NOM).

The study aims to present current advances and controversies in LARC patient management and clarify the role of TNT in current management from the perspective of indications, neoadjuvant treatment efficacy, response assessment methods, surgical treatment importance, and the WW strategy. The goal is to assist physicians in selecting the optimal, patient-centered strategy by balancing medical criteria, patient preference, and healthcare provider capabilities.

## 2. Materials and Methods (See Table 1 and Table S1)

We conducted a systematic literature review in Medline/PubMed using various keyword combinations, including “rectal cancer/neoplasia” and “therapy”or “neoadjuvant therapy” or “TNT” (Table S1). We included articles published between 2015 and 2025, and the search strategy is presented in Table 1.

**Table 1:** The search strategy summary.

Items	Specification
Database and other sources searched Search terms used (including MeSH and free text search terms and filters) Timeframe Inclusion and exclusion criteria (study type, language restrictions, etc.). Selection process	PubMed Central (PMC) Search strategy (see Table S1) 2015–2025 Inclusion criteria: meta-analyses; trials studies; clinical trials & updates of clinical trials; reviews; original articles; only studies/papers/journals written in English. Exclusion criteria: unpublished data from abstracts contained in volumes from various congresses or conferences; papers that were not in English RDB performed the search in the databases according to the presented criteria. RDB conducted the database search based on the specified criteria. A total of 234 records were identified using the advanced search builder with the applied filters (Table S1). Of these, 54 articles were excluded using automation tools. The remaining 180 articles were screened by two independent reviewers, RDB and RES, resulting in the exclusion of 25 based on title and abstract. The remaining 155 reports were assessed for eligibility. If a study was deemed irrelevant by either RDB or RES, the full text was reviewed by a third reviewer (MG) for final evaluation. As a result, 15 reports were excluded. Additionally, the reference lists of the included articles were examined for further relevant studies, and 82 additional records were identified through citation screening (Figure 1).

**Figure 1:**
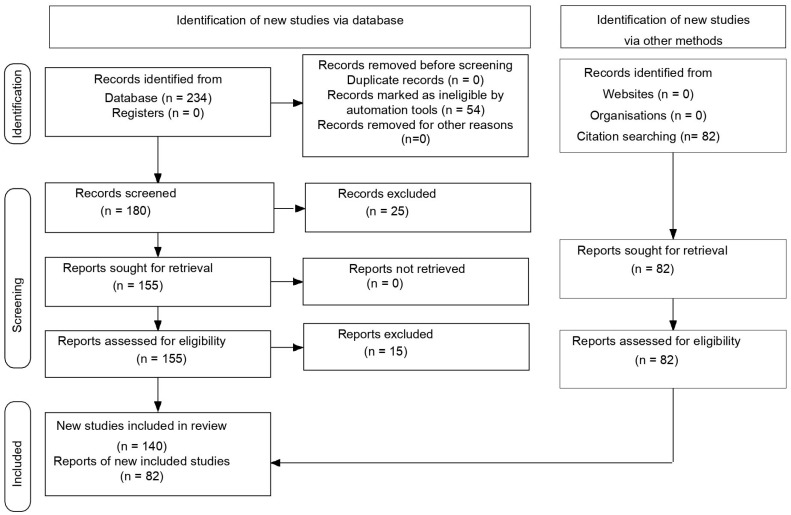
PRISMA flow chart of study.

## 3. Therapeutic Options over Time

Traditionally, RC was treated surgically via abdominal or abdominoperineal resection—Miles’ procedure. Only unresectable tumors were referred for complementary treatment (RT/CT). The poor prognosis of RC due to high LR rates and distant metastases led to the adoption of alternative strategies to improve outcomes.

A new surgical approach became necessary, based on the premise that LR results from residual tumor tissue within the mesorectum. This led to the introduction of total mesorectal excision (TME), which is now the gold standard for surgical resection. In 1986, Heald and colleagues published results on TME [15], showing a 5-year LR rate of 3.7%, which decreased further to 0.5% in subsequent years among patients undergoing curative resections [16].

One of the first randomized studies, published in 1986, demonstrated a reduction in 5-year LR in patients with advanced stage B2 and C tumors undergoing radical surgery—from 55% in those treated with surgery alone to 33% in those receiving adjuvant radiochemotherapy through RT and 5FU as postoperative treatments [17].

In the following decade, several studies [18,19] confirmed that using S-RT reduced LR rates, and applying neoadjuvant S-RT resulted in significantly lower LR rates compared to adjuvant S-RT [20].

Other randomized clinical trials demonstrated that neoadjuvant S-RT followed by TME was more effective than TME alone [21,22]. The LR rate was three times higher in the surgery-only group (26%) compared to the neoadjuvant treatment group (9%, *p* < 0.001) [23].

In LARC patients, neoadjuvant CRT reduced the 5-year LR rate to 6% compared to 13% in the adjuvant CRT group, but without benefits for OS, DFS, or distant metastases [24].

Although neoadjuvant CRT can achieve a complete pathological response (pCR) in approximately 20% of LARC patients, a significant proportion respond only partially or not at all [25,26], requiring alternative therapeutic approaches.

Some studies report that neoadjuvant CRT improves OS and local disease control, particularly in patients with pCR [27,28]. Thus, neoadjuvant CRT followed by TME has become the treatment of choice for LARC [29], with LR rates below 10% [16].

TNT integrates CT and RT preoperatively to enhance adherence, timing, and systemic control. Clinical trials such as RAPIDO, OPRA, and PRODIGE 23 have demonstrated theirsuperiority in tumor downstaging and improving cCR or pCR. Additionally, organ preservation strategies, such as WW, have emerged as viable options for patients achieving a complete response to neoadjuvant therapy [30].

## 4. Current Indications for Total Neoadjuvant Therapy

### 4.1. TNM Stage—T3/T4N+

TNT has emerged as a viable option for patients with LARC at risk of metastases and LR [31].

Some authors suggest that TNT is appropriate for certain patients with stage II/III LARC, stages that generally have an indication for neoadjuvant therapy [32]. For T3N1 tumors, TNT may be recommended only in the presence of additional risk factors. The indication for TNT in patients with T3N0 tumors is debatable; some studies showed that these patients do not require TNT but only neoadjuvant CRT [33]. Other studies have shown that patients with T3N0 rectal tumors may benefit from TNT, especially if an organ-preserving strategy is desired. Moreover, these patients have the highest chances of achieving a complete response after TNT and maximizing the benefits of this strategy [34].

The National Comprehensive Cancer Network (NCCN) guidelines recommend TNT for patients with inadequate performance status for surgery, unresectable tumors, T3 tumors with positive circumferential resection margins (CRM), T4 tumors, or positive lymph nodes [35]. The presence of high-risk features has been the primary eligibility criterion for pivotal TNT studies. Imaging characteristics of patients included in the RAPIDO trial were as follows: T4, N2, extramural vascular invasion (EMVI) positive, mesorectal fascia (MRF) involvement, or enlarged lateral pelvic lymph nodes [36].

In the PRODIGE-23 study, patients with stage II–III rectal cancer were included regardless of the presence of risk factors. Approximately 90% of the patients had cN+ tumors, a small proportion had T4 tumors (17% vs. 31%), and MRF involvement was lower (27% vs. 61%) compared to the RAPIDO study [37].

A recent study suggests that for patients with tumors at high risk of recurrence or distant metastases, TNT appears to be a logical option. However, the selection criteria for TNT remain debatable, and it should not be applied to all LARC patients. Some authors have introduced the “good–bad–ugly” concept, identifying an intermediate group consisting of less advanced cT3–4 or N+ tumors.The distinction between the “bad” and “ugly” groups is based on their respective risk levels for LR. Most cT4 tumors are classified in the “ugly” group, as well as those with MRF involvement or signs of lateral node involvement [38].

High-quality imaging can identify patients with a very low risk of LR, allowing for the omission of neoadjuvant RT in these cases. The criteria for optional RT omission (T3 tumors with a maximum infiltration of 5 mm into perirectal fat, without clearly affected lymph nodes [39]), may be expanded based on data from the PROSPECT [40] and OCUM [41] studies.

In the PROSPECT trial, tumors classified as T2, N1, or T3, regardless of nodal involvement, were included, provided that the distance to the CRM was at least 3 mm and sphincter-preserving surgery was feasible [39]. In the OCUM study [41], patients with LARC (cT2-4, any cN, cM0) were treated differently based on LR risk. The risk assessment considered the distance to the CMR, suspicious lymph nodes or tumor deposits, and MRF involvement. Patients with a distance >1 mm underwent direct TME (low-risk group), while those with a distance ≤1 mm and/or cT4 tumors and cT3 tumors in the lower rectal third received neoadjuvant CRT followed by TME (high-risk group). In both studies (PROSPECT and OCUM), the LR rate was below 3%.

### 4.2. Contraindications for TNT

KRAS and p53 mutations occur in approximately 40–70% of rectal tumors and are associated with a poor response to neoadjuvant CRT [42,43]. Approximately 5% of RC are microsatellite instability/mismatch repair deficiency (MSI/MMRd), which confers resistance to 5FU [44], but they show a good response to immunotherapy with immune checkpoint inhibitors. TNT based on CT is not recommended for patients with MMRd/MSI rectal cancer [45].

In addition to the standard testing for RAS and BRAF mutations, as well as for MSI/MMRd status, genotyping of patients for dihydropyrimidine dehydrogenase (DPYD) should also be introduced [46]. Patients with DPYD deficiency present with increased toxicity to fluoropyrimidines, and in these cases, the discontinuation of TNT should be considered.

## 5. Radiotherapy in LARC

Neoadjuvant RT is an effective method to improve local control of LARC by reducing tumor size and achieving tumor downstaging. LCRT may be the best choice for locally advanced tumors (T4, MRF+), particularly for patients with borderline WHO performance status who may not be able to complete the full course of combined neoadjuvant CT. The advantages of this therapy include high R0 resection rates and sphincter preservation rates in cases of low rectal tumors [24], as well as low rates of LR [47,48]. It is also preferred for patients undergoing the WW strategy. The OPRA study used LCRT for patients with T4b tumors, with invaded MRF, or those with low rectal tumors. More than 70% achieved a complete or near-complete clinical response and followed the WW strategy [49]. On the other hand, it is not recommended as an initial therapy for patients with a very high risk of distant metastasis or those already showing evidence of metastases due to its limited systemic effect.

S-RT has demonstrated a significant reduction in LR in several phase III studies [18,50]. However, S-RT has been associated with lower tumor response rates due to the short interval between RT and surgery. The Stockholm III study confirmed the downstaging effect of delaying surgery [51], as did several subsequent studies [52,53].

The issues with S-RT are related to the risk of late LR, which is more common compared to CRT, although the S-RT regimen has not been shown to be inferior to CRT, especially when combined with sequential systemic CT. In the TNT strategy, S-RT can be a better option when administered prior to CT for patients at high risk of systemic disease, as demonstrated by the RAPIDO study. A pathologic complete response (pCR) rate of 28% was observed in patients in the TNT group with S-RT, compared to 14% in the CRT group [36]. However, a higher rate of LR was detected after S-RT (10.2%) compared to the CRT group (6.1%) [14]. This study is the most controversial due to the higher LR rate in the S-RT group [54,55].

Several studies comparing S-RT followed by CT with standard CRT have shown that at least four cycles of CT should be administered to achieve higher rates of pCR and disease-free survival (DFS) [56].

Other authors have found no differences in terms of LR, surgical complications, and OS between the two types of RT [57,58]. S-RT has not been used after neoadjuvant CT in TNT studies, so there is no evidence supporting this treatment sequence [37,49,59].

S-RT has certain advantages: an approximately one-month shorter treatment duration, rapid symptom remission, and studies like POLISH II [60] and ESCORT [61] have shown it to be more cost-effective in terms of both direct and indirect costs compared to standard CRT.

Some authors have described the possibility of intermediate-intensity RT, but the experience is limited. The KROG 11-02 study examined the utility of this type of RT in patients with T3-4/N+ tumors, using an intermediate regimen of 33 Gy/10 fractions, administered concomitantly with capecitabine. No significant differences were found compared to CRT in terms of pCR rate (ypT0N0—16.7% vs. 23.3%), tumor regression grade based on the Dworak score (16.7% vs. 23.3%), or 5-year OS [62].

Higher radiation doses were abandoned due to significant adverse effects. According to the Swedish Rectal Cancer study, where patients were treated with high-dose RT, the long-term effects included fecal incontinence, urgency, and difficulty in evacuation. Thirty percent of these patients reported an impairedsocial life due to bowel dysfunction, compared to 10% among those who did not undergo RT [63].

The late side effects of neoadjuvant S-RT included fecal incontinence, rectal bleeding, mucus loss, urinary incontinence, and increased cardiovascular morbidity, which were more frequent than in patients treated with only surgical TME [64,65]. Lastly, hematological toxicity due to pelvic bone marrow irradiation, as well as infertility and early menopause, have been described, which are particularly important concerns for younger individuals [66,67].

Since the effect of RT on OS is limited, systemic recurrence remains the primary issue for patients with LARC, with 25% developing distant metastases during follow-up [68,69]. Therefore, the addition of systemic CT has been proposed as part of the complementary treatment.

## 6. The Importance of Chemotherapy in the Management of LARC

### 6.1. 5FU and Oxaliplatin

Significantly superior results with neoadjuvant CRT compared to neoadjuvant RT as monotherapy have been observed in the FFCD9203 [70] and EORTC22921 [71] studies, which improved the pathological complete response (pCR) rate to 11.4–14% and the 5-year LR rate to 7.6–8.1%.

Following the results published by the German study of Sauer et al. [24], the use of preoperative 5FU concomitant with LCRT followed by TME and postoperative adjuvant 5FU therapy became the standard treatment for LARC, at least in Europe [72].

5FU is a radiosensitizing agent that enhances tumor response but must be administered via continuous infusion during RT. This inconvenience was addressed by introducing the orally bioavailable fluoropyrimidine-capecitabine (Xeloda^®^). Large phase III studies [73] have shown that it has similar efficacy and safety to 5FU in RC patients. Other studies have demonstrated that the standard doses of capecitabine or 5FU have no effect on distant micrometastases and do not improve OS [74,75].

Oxaliplatin blocks DNA replication and transcription and has a higher radiosensitization capacity than 5FU [76], with which it acts synergistically [77]. Its better efficacy in metastatic colorectal cancer therapy or as adjuvant therapy [78] led to its use in conjunction with neoadjuvant RT in patients with LARC to increase downstaging rates and improve OS [79]. However, the additional benefit of oxaliplatin remains controversial.

All phase III clinical studies, except one, have demonstrated increased toxicity without clinical benefits for oxaliplatin as concomitant CT in CRT. In the STAR-01 [80] and FOWARC [81] studies, oxaliplatin was associated with 5FU and RT, while in the ACCORD 12/0405 PRODIGE 2 [82], NSABP R-04 [83], JIAO (Liaoning Cancer Hospital) [84], and PETACC-6 [85] studies, capecitabine replaced 5FU/LV. The addition of oxaliplatin did not impact the primary tumor response, LR rates, DFS, or OS compared to patients receiving only 5FU/LV plus RT. The phase III CAO/ARO/AIO-04 study showed that the addition of oxaliplatin improved the pCR rate (17% vs. 13%) and 3-year DFS (75.9% vs. 71.2%) with similar rates of grade 3 and 4 adverse events [86].

Other authors have noted that in patients aged over 70–75 years and/or with major comorbidities, oxaliplatin-based CT is associated with increased toxicity and thus reduced benefit compared to 5FU-based CT alone [87].

Even though the introduction of oxaliplatin has not always demonstrated a clear benefit and sometimes resulted in greater toxicity [80], the potential benefits shown in the CAO/ARO/AIO-04 study have driven its use with 5FU or capecitabine during RT as part of neoadjuvant or adjuvant treatment for patients with LARC. Since 2018, the American NCCN guidelines recommend this strategy as the standard of care for these patients [88].

### 6.2. The Role of Adjuvant Chemotherapy (CT)

Despite the favorable effects on LR and OS rates obtained with adjuvant CT in colon cancer, it has not sufficiently improved outcomes in RC. Some authors have noted slight improvements in patients with primary surgery [89], but other studies have not demonstrated its efficacy in patients with neoadjuvant treatment [90].

The ADORE study investigated the use of adjuvant 5FU/LV compared to FOLFOX. There was no difference in 6-year OS, but FOLFOX improved DFS [91]. Another study demonstrated that adjuvant CT with 5FU/LV improved DFS and LR in patients with low rectal tumors <10–15 cm from the anal margin, compared to those with tumors <10 cm from the anal margin [92].

According to the current guidelines of the NCCN, for stage II or III patients, adjuvant CT is recommended for 6 months with or without neoadjuvant treatment, regardless of the pathological regression response [35]. However, its impact on OS and DFS in these patients is controversial. Some studies suggest that adjuvant treatment may improve OS and DFS in LARC [93], while others argue that it does not affect the oncological prognosis of patients receiving neoadjuvant therapy [94]. It is noteworthy that in patients enrolled in many randomized controlled trials, adjuvant therapy was not in line with NCCN recommendations but was left to the discretion of the oncologist [95].

Despite the recognized prognostic advantage of pCR, the necessity of adjuvant treatment in these patients remains uncertain. According to the guidelines of the European Society for Medical Oncology (ESMO), adjuvant treatment is recommended after neoadjuvant therapy only for stage II/III tumors with high-risk factors [31].

Several cohort studies have retrospectively analyzed the prognosis of patients with pCR with or without adjuvant therapy. Recent results have shown that adjuvant treatment improves OS in patients with pCR [96,97], in contrast to other authors who did not observe this effect [98].

The poor efficiency may be explained by poor compliance with adjuvant treatment [90]. Perioperative complications or the presence of a protective ileostomy can cause delays, de-escalation, early discontinuation, or prevent the administration of CT. Thus, only 25–50% of patients completed adjuvant CT, compared to 80–90% of patients who received it as part of the neoadjuvant sequence [99]. On the other hand, up to 30% of patients suffer from distant relapse at 5 years [100]. Given the debated role of adjuvant CT, administering the entire systemic treatment before surgery seems to be an appealing approach.

### 6.3. TNT vs. Standard CRT

The concept of TNT involves the use of either S-RT or LCRT and the advancement of adjuvant CT as part of the neoadjuvant treatment [101]. TNT may reduce the risk of distant metastasis and increase the pCR rate [102], allowing for the application of organ-preserving strategies in selected patients. Combined CT is the cornerstone of TNT, representing the main advancements of TNT over standard neoadjuvant CRT. Phase III clinical studies of TNT have reported more significant tumor regression and a reduced incidence of distant metastases [103]. In three phase III studies (RAPIDO [36], PRODIGE [37], STELLAR [58]), the superiority of TNT over standard CRT was demonstrated, achieving a pCR rate of 27.8–28% and an LR rate of 4.8–8.7%. Combined CT regimens were administered for 3–4 months (6 cycles of CAPOX or 6–9 cycles of FOLFOX/FOLFOXIRI).

### 6.4. Neoadjuvant Induction vs. Consolidation Chemotherapy

Multi-agent systemic CT regimens, such as infusional 5-FU, leucovorin (LV), and oxaliplatin (FOLFOX) or 5FU, oxaliplatin, and irinotecan (FOLFOXIRI), can be administered before RT as induction chemotherapy (INCT) or after RT as consolidation chemotherapy (CNCT). Both sequences improve outcomes compared to conventional preoperative CRT with or without adjuvant CT [36,37].

The randomized CAO/ARO/AIO-12 study included patients in group A with INCT and group B with CNCT, followed by TME in both groups. CT consisted of FOLFOX for three cycles, while CRT was associated with 5FU and oxaliplatin. CNCT led to better compliance with RT (97% vs. 91%) but lower compliance with CT compared to INCT (85% vs. 92%). However, the pCR rate was higher in the CNCT group (25%) compared to INCT (17%) (*p* < 0.001). No differences were found regarding DFS or LR rates, or the incidence of distant metastases at a 3-year follow-up [59,104].

The OPRA study enrolled patients with LARC who were treated with INCT or CNCT, followed by either TME or organ-preserving management. Patients in both groups received four months of FOLFOX or CAPOX and RT (50–56 Gy) associated with 5FU or capecitabine. Similar results were observed for DFS, OS, estimated survival rates without LR, and survival rates without distant metastases at 3 and 5 years [105]. Five-year OS in patients without TME was higher after CNCT (54%) compared to INCT (39%) [106].

Studies to date have suggested that CNCT is more effective because it allows more time for rectal tumors to respond to RT. CNCT regimens using 5FU with or without oxaliplatin have demonstrated increased rates of clinical complete response (cCR) and organ preservation. However, the benefit of adding oxaliplatin regarding primary tumor response remains unclear. To incorporate oxaliplatin into standard CNCT regimens, this benefit needs to be clarified due to its considerable toxicity [107].

### 6.5. Strategies with Intensified Neoadjuvant Chemotherapy

To achieve pCR rates over 20% (which have not been reached using CRT in LARC) or complete response (CR) rates over 40–50%, it may be necessary to administer 3–4 months of CT after S-RT [35,58], whereas 1–2 months is insufficient [108].

The UNICANCER-PRODIGE 23 study [37] demonstrated that survival outcomes improve with intensified neoadjuvant CT. Intensified CT (FOLFOXIRI × 6 cycles → CRT → TME → FOLFOX for 3 months) significantly improved pCR from 12% to 28% and 3-year progression-free survival (PFS) from 62% to 69%. The updated report showed better long-term results after INCT: OS (81.9% vs. 76.1%), DFS (67.6% vs. 62.5%), and cancer-specific survival (84.9% vs. 79.6%). This regimen eliminates early-stage micrometastases and improves patient compliance with treatment.

These results led to the hypothesis of using combined neoadjuvant CT as monotherapy, thereby eliminating the long-term side effects caused by RT. The FOWARC study compared the results of three strategies: mFOLFOX CT pre- and postoperatively, standard CRT followed by TME and adjuvant 5FU therapy, and INCT FOLFOX followed by LCRT and TME with adjuvant FOLFOX. Neoadjuvant monotherapy with mFOLFOX6 resulted in a pCR rate of 6.6%. Rates of R0 resection (89.4% vs. 90.7%), anal sphincter preservation (89.5% vs. 84.4%), and 3-year LR (8.3% vs. 0%) were similar to those seen with standard CRT followed by TME and adjuvant 5FU therapy, with fewer postoperative complications. Although no significant differences were found in 3-year DFS (72.9%, 77.2%, 73.5%), adding RT improved the pCR rate (14.0%, 27.5%, 6.6%) and tumor downstaging (37.1%, 56.4%, 35.5%) [109].

The PROSPECT study compared neoadjuvant FOLFOX administration with CRT, applied only if the tumor did not shrink by at least 20% after CT. In approximately 90% of patients treated with FOLFOX, CRT was not applied. Patients in the surgical treatment group showed similar results in the FOLFOX and CRT groups regarding pCR (21.9% vs. 24.3%), 5-year OS (89.5% vs. 90.2%), and 5-year LR (1.8% vs. 1.6%) [40,81].

The GRECCAR-4 study [105] initially treated patients with FOLFOXIRI. Of the 30/133 patients with a good response, they were randomized to surgery or CRT followed by surgery. A group of patients with chemotherapy-sensitive tumors was selected, and CRT was omitted. This aspect may support starting with CT, although this sequence had poorer results in two studies: CAO/ARO/AIO-12 and OPRA.

Another study evaluating neoadjuvant XELOXIRI without CRT found acceptable LR and DFS rates, but the pCR rate was 7.7%, which was lower than expected [110]. Other authors observed a higher pCR rate in the irinotecan group (30% vs. 15%) and a trend toward improved DFS and OS, but with more frequent grade 3–4 toxicities [111]. In contrast, the phase III ARISTOTLE study showed that adding irinotecan did not improve the pCR rate, but it increased the rate of adverse events and substantially reduced treatment compliance [112]. Therefore, the value of adding irinotecan to neoadjuvant CRT for RC remains unclear.

### 6.6. The Role of Targeted Therapy in Patients with LARC

While the overall impact of systemic CT may seem promising, there is room for further improvements. It is conceivable that more aggressive regimens, combining CT with anti-angiogenic agents or monoclonal antibodies targeting epidermal growth factor receptor (EGFR), could lead to higher rates of tumor regression, including in patients with poor responses to CRT [113,114].

The TRUST study investigated the use of FOLFOXIRI and bevacizumab (anti-VEGF agent) as neoadjuvant CT (INCT), followed by CRT with capecitabine and bevacizumab concurrently, followed by surgery at 8 weeks in patients with LARC. The pCR rate was 36.4% and the 2-year DFS was 80.45% [115]. Adding bevacizumab to CRT or TNT may enhance the treatment response, but it also increases toxicity, especially in terms of wound healing and the development of fistulas, necessitating its discontinuation one month before and after RT or surgery [116].

EGFR overexpression has been reported as a predictive factor for resistance to RT in RC, so using anti-EGFR agents may be a strategy for these patients [117]. Anti-EGFR agents such as cetuximab or panitumumab are effective only in wild-type RAS and BRAF tumors [118], significantly improving the response rate [119]. Another phase II study, the EXPERT-C Trial, evaluated adding cetuximab to TNT. Although there was no difference in complete response rates or PFS in patients with wild-type KRAS, significant improvements were observed in radiological response after CT (51% without cetuximab vs. 71% with cetuximab, *p* = 0.038) and CRT (75% without cetuximab vs. 93% with cetuximab, *p* = 0.027), as well as OS (HR 0.27, *p* = 0.034) [120].

In the phase I/II VOLTAGE-A study, CRT followed by 5 cycles of nivolumab led to a pCR rate of 33% in patients with microsatellite stable (MSS) tumors, reaching 60% in patients with microsatellite instability-high (MSI-H) tumors [121]. In the phase II AVANA study, CRT followed by six cycles of avelumab achieved a pCR rate of 23% [122]. Another study showed a pCR in 25% of patients and near pCR (TRG-1) in 25% using S-RT followed by intensified neoadjuvant CT (six cycles of mFOLFOX6 plus avelumab) [123]. Other authors reported a pCR rate of 48.1% using two cycles of CAPOX plus camrelizumab [124].

While these studies enrolled patients without distinguishing MMR status, it is important to note that MSS patients benefit more from immunotherapy. Approximately 5–10% of rectal adenocarcinomas have deficient mismatch repair (dMMR), and these tumors respond poorly to standard CT regimens [125].

The Keynote 177 study demonstrated a benefit in PFS (median, 16.5 vs. 8.2 months; HR, 0.60; 95%CI, 0.45–0.80; *p* = 0.0002) with the addition of pembrolizumab in patients with MSI-H/dMMR metastatic RC [81]. Similar results were observed in the Checkmate 142 study, where the combination of nivolumab achieved an objective response rate of 69% and a CR rate of 13% [109].

Another study investigated the overall response and sustained cCR rate with neoadjuvant treatment using dostarlimab, a PD-1 inhibitor. Twelve patients completed treatment with dostarlimab and had at least six months of follow-up, all showing cCR (100%; 95% CI, 74–100) [44].

The 2024 NCCN guidelines recommend checkpoint inhibitor immunotherapy for up to six months (dostarlimab-gxly, nivolumab, or pembrolizumab) in patients with LARC with MSI-H/dMMR and advise identifying MSI status to offer personalized treatment [123].

## 7. Evaluation of Response to Neoadjuvant Treatment

Approximately 20–30% of patients with LARC who receive neoadjuvant CRT achieve a complete clinical or pathological response. However, a significant number of patients do not respond to CRT [25]. Therefore, an important role in managing LARC is the identification of markers to predict response to neoadjuvant CRT. The association between tumor distance from the anal verge and response to neoadjuvant chemoradiotherapy (CRT) as a predictive marker remains unclear [126]. Clinical T and N staging (cT and cN) are not sufficient for prediction, although it has been reported that T and N stages influence the response to neoadjuvant CRT [127].

The prediction of response to treatment is assessed using magnetic resonance imaging (MRI). MRI has become a necessary standard for evaluating locally advanced disease, particularly in patients with potential involvement of CRM. The macroscopic spread of the tumor beyond the muscularis propria is associated with a worse prognosis. Tumor spread of less than 5 mm is associated with a prognosis similar to that of T2 tumors, as long as there is no involvement of the CRM. Tumor spread greater than 5 mm into the mesorectum (T3c-d) is associated with increased rates of disease recurrence [128].

It has been shown that MRI accurately identifies the potential distance from the tumor to the CRM margin of 1 mm, which is recognized by pathologists as a clear margin [129]. The results of the MERCURY prospective study showed that the specificity of MRI for predicting pCRM is 92% [130].

According to the TNM classification system, tumors invading the MRF are not classified as T4—therefore, the MRI-predicted CRM status should always be reported in addition to TNM. A measured distance of 1 mm or less from the primary tumor, tumor/vascular deposit, or invaded extramural vessels to the MRF is one of the major prognostic factors for reduced LR, DFS, and OS.

Traditionally, extramural venous invasion (EMVI) is evaluated on the resected specimen (pEMVI), and it is an independent predictor of LR, distant metastases, and OS [131]. Currently, MRI identifies the preoperative EMVI status (mrEMVI), guiding patient management [132].

It has been shown that EMVI, defined as the presence of tumor signal within the vascular system outside the muscularis propria, correlates with histopathological results [133] and survival outcomes [134]. Its presence in low rectal cancers is associated with a risk of CRM involvement and LR [135].

pCR is characterized by the complete absence of residual tumor cells both at the primary tumor site and in the mesorectal lymph nodes [136]. The degree of tumor regression (TRG) is assessed on the specimen and reflects the response to neoadjuvant therapy. It is based on the evaluation of the volume of residual tumor cells (Ryan, Dworak, and Mandard) and has demonstrated prognostic ability for DFS and OS [137]. Additionally, ypT, ypN, and yp Stage are prognostic factors [138], with ypN having the greatest prognostic ability [139].

Even though histopathology remains the so-called gold standard for evaluating tumor response in the final specimen, the opportunity to modify treatment before definitive surgery is often missed. Thus, preoperative identification of the treatment response allows for appropriate patient selection for either surgical treatment or the WW strategy. To evaluate clinical response after neoadjuvant treatment, the following are used: digital rectal examination (DRE), endoscopy, and MRI (T2W and DWI sequences). All three modalities combined have an accuracy of 98% in predicting the absence of the tumor [140].

Thus, there are three types of responses: complete clinical response (cCR), nearly complete clinical response (ncCR), and incomplete response. A typical cCR appears as a flat, white scar on endoscopy, without ulceration or nodularity, with signs of fibrosis only on DRE and MRI [141].

Definition of complete clinical response: DRE: mucosa without irregularities, firm area with minor induration, endoscopic: white scar, telangiectasia, absence of ulceration and/or tumor mass [142], MRI: mrTRG1: fibrosis with low signal intensity on T2-weighted images replacing the primary tumor; no restricted diffusion on diffusion-weighted images; no nodes with border irregularity or mixed signal intensity; no EMVI [143].

Definition of nearly complete response: DRE: superficial ulceration or minor irregularities (doubtful) of the mucosa/rectal wall, endoscopic: residual tumor size ≤ 2 cm (or ≥70% reduction in the volume/size of the original tumor) [144,145], MRI: mrTRG2: predominance of fibrosis with low signal and foci of intermediate signal intensity from the tumor visible on T2-weighted images with or without restricted diffusion; mrTRG1: fibrosis with low signal intensity visible on T2-weighted images replacing the primary tumor with restricted diffusion; no node with border irregularity or mixed signal intensity; no EMVI [145].

Some authors [146] have described the “split scar” sign (SSS) in restaging MRI: a regular hypointense scar indicating fibrosis in the submucosa on T2-weighted images, with an underlying layer of intermediate signal intensity at the muscularis propria level and a third outer hypointense layer corresponding to perirectal fibrosis. An interruption of the hypointense scar by an intermediate signal on T2-weighted images indicates a residual or recurrent tumor. Although the initial study reporting SSS showed a specificity of 97% in identifying complete responses, subsequent studies have demonstrated variable diagnostic performance [147,148].

Patients whose initial lymph node and EMVI statuses were positive but later negative after neoadjuvant treatment had DFS and OS similar to those of patients who were initially EMVI-negative and better compared to those who remained positive after treatment [149]. Therefore, identifying post-treatment EMVI has long-term prognostic significance for patients with complete response.

Other authors have observed 100% concordance of restaging MRI after neoadjuvant treatment with pCR [150]. The use of all three modalities could predict the absence of tumor cells with an accuracy of 98% [140].

According to a study evaluating metabolic characteristics detected by [18F] FDG-PET/CT before CRT, the baseline parameters of [18F] FDG-PET correlated with tumor staging and tumor metabolic volume have been correlated with the TRG score, but without prognostic ability [151]. The Habr Gama team demonstrated that a variation of more than 76% in the standard uptake value (SUV) between the initial PET scan and the one performed 12 weeks after CT and RT was significantly associated with cCR. However, PET scanning was considered less reliable (85% of cases correctly classified) than clinical evaluation (91% of cases correctly classified) [152]. Other authors suggest that early SUV variations are highly predictive of a complete response [153].

Studies have demonstrated that both PET scanning and diffusion-weighted MRI can differentiate between non-responsive and responsive tumors, but both methods remain imprecise in identifying complete responses [154].

cCR is used as a surrogate marker for pCR in clinical trials evaluating the response to neoadjuvant CRT for LARC [127].

Analysis of circulating DNA via liquid biopsy, to evaluate the response to CRT, could be an important factor in adjusting therapy and monitoring patients [155]. Some authors have observed that circulating DNA status may anticipate pCR during multimodal treatment [136]. The preoperative detection of circulating DNA (GEMCAD 1402 study) [156] or at 4 weeks post-surgery (GALAXY study) has been associated with an increased rate of systemic recurrence, reduced DFS, and OS [157].

Biopsy for residual mucosal abnormalities and additional imaging, such as PET-CT, are not routinely recommended [140].

Other studies show that up to 50% of patients achieve a cCR and do not require surgery [105,158]. Thus, NOM is an option for selected, well-informed patients willing to follow an intensive follow-up regimen. Patients with a complete or nearly complete clinical response 4–8 weeks after finishing TNT can enter this program [159].

Currently, it is recommended that during the first 3 years after TNT, surveillance should include CEA every 3 months; DRE, rectoscopy, and pelvic MRI every 3–4 months; and CT scans of the chest and abdomen every six months. After 4 years, evaluations can be performed using serum CEA, DRE, rectoscopy, and pelvic MRI every 6 months, alongside CT scans of the chest and abdomen every 12 months [160].

The rate of LR is estimated at 20–40% and is most commonly observed in the first 2 years, treated through radical surgery without compromising DFS [106].

## 8. Effectiveness of TNT

Several studies support the improved effectiveness of TNT compared to the standard approach, particularly regarding pCR, LR rates, and DFS [14,161]. Recent data suggest a potential improvement in OS. Patients with cCR after TNT have the option to opt for NOM.

The main concern in the concept of TNT is the delay in radical surgical intervention, which could lead to an extension of the primary tumor, invading local structures or spreading metastatically in the case of tumor resistance to neoadjuvant therapy [106].

Studies have not shown differences in the proportion of patients undergoing radical surgery (over 90%), the type of surgery, R0 resections, and the quality of TME when comparing TNT and standard treatment. Although tumor downstaging is evident after TNT, progression and metastatic spread may still occur. However, in the majority of these cases, distant micrometastases are already present at the time of diagnosis and only manifest later in the course of the disease [162].

TNT can improve treatment effectiveness by increasing compliance with preoperative CT, initiating the early treatment of subclinical metastatic disease, and increasing the response rate of the primary tumor. One study noted that 85% of patients with TNT completed CT, compared to 67% of patients with standard treatment [163].

The shorter total duration of TNT, approximately 1–2 months less than standard treatment, favors better treatment compliance since this period is associated with considerable limitations in the patient’s professional and personal life [99].

### 8.1. Markers of Neoadjuvant Treatment Effectiveness

Therapeutic effectiveness is evaluated in terms of short-term results(clinical or pathological tumor response) and long-term outcomes (DFS at 3 and 5 years and OS). The relationship between these is also crucial for reporting long-term results using immediate outcomes.

Extensive research has shown that patients with pCR have remarkably low LR rates (6–17%) and high y5OS rates (87–92.9%) [164,165]. A meta-analysis showed that patients with pCR have better DFS and OS than those without pCR [166]. Therefore, pCR has been used as an endpoint in the design of clinical trials and as a surrogate marker for long-term prognosis, although some authors argue that it is a weak surrogate for OS [167], and a meta-analysis [168] shows that DFS is a stronger predictor of OS than pCR.

Other authors have shown that complete clinical response (cCR) of the tumor was associated with DFS, survival without LR, survival without distant metastases, and OS [169]. Of course, the correlation between cCR and pCR is not perfect, as it has been observed that up to 25–30% of patients with cCR continued to progress [170], mostly in the first 2 years of follow-up.

### 8.2. Rate of pCR

After standard CRT for T3/T4 rectal cancer, only 15% of patients exhibit cCR and are eligible for NOM [158,171].

Strategies that can be used alone or in combination to improve this rate include increasing the dose of neoadjuvant RT, prolonging the interval between neoadjuvant treatment and surgery, and adding neoadjuvant systemic CT to RT [104].

A report encompassing nearly 3300 patients indicated that the neoadjuvant radiotherapy (RT) dose was a predictor of pCR [172]; however, it remains uncertain whether this approach enhances the rate of NOM. Other studies suggest that the local response improves over time following RT [105].

The TNT strategy has been shown to double the cCR rates compared to CRT, making it an attractive approach for NOM in approximately one-third of patients with distal tumors [36,99]. In the CAO/ARO/AIO-12 study, the addition of four cycles of FOLFOX either before or after standard CRT resulted in pCR rates of 17% for induction chemotherapy (INCT) and 25% for consolidation chemotherapy (CNCT), respectively [59].

A meta-analysis confirmed that pCR rates are superior following various TNT regimens—such as long-course RT (LCRT) or short-course RT (S-CRT) plus CNCT, or INCT plus LCRT—compared to standard CRT [173]. Similar outcomes have been observed by other researchers; both CNCT (odds ratio [OR] 2.36; 95% confidence interval [CI], 1.57–3.66) and INCT (OR 1.99; 95% CI, 1.44–2.95) significantly improved complete response (CR) rates, with CNCT demonstrating the highest efficacy in achieving CR (surface under the cumulative ranking curve [SUCRA] 0.90) [174].

### 8.3. Local Recurrence Rate

A less desirable aspect is that the LR rate after TNT is not negligible. The RAPIDO study observed an almost twofold increased risk of LR in patients undergoing TNT compared to standard therapy (6.3% vs. 3.8%), with this difference persisting over time and eventually reaching statistical significance (10% vs. 6%; *p* = 0.027) [14]. These findings have raised concerns regarding the use of S-CRT. However, the subsequent STELLAR study did not demonstrate differences in LR rates (8.4% in the TNT group vs.11% in the standard treatment group) and reported a significantly higher overall CR rate in the TNT group (21.8%) compared to the standard treatment group (12.3%, *p* = 0.002) [58].

### 8.4. Distant Survival

The implementation of TNT in the PRODIGE23 and RAPIDO studies demonstrated an improvement in DFS; however, regarding OS, only the PRODIGE23 study succeeded in demonstrating a benefit. In PRODIGE23, TNT with INCT-CRT using FOLFOXIRI followed by mandatory total mesorectal excision (TME) and adjuvant FOLFOX led to a significant improvement in 3-year DFS compared to the standard of care (76% vs. 69%; *p* = 0.03). A 7-year follow-up reported superior DFS (67.6% vs. 62.5%; *p* = 0.048) and OS (81.9 vs. 76.1 months, *p* = 0.033) [175].

Additionally, two randomized studies observed similar DFS outcomes for both therapies, but CNCT resulted in a higher rate of NOM [106]. Similar findings were observed in the OPRA study, which employed TNT regimens with multiple chemotherapy cycles and offered the NOM strategy to patients with a cCR or near-complete response (nCR) [107]. In the CAO/ARO/AIO-12 study, with a median follow-up of 5.1 years, survival without surgical treatment was 39% with INCT and 54% with CNCT (*p* = 0.012), with similar 5-year DFS rates (71% and 69%; *p* = 0.68) [176].

Other authors have shown that S-RT followed by CNCT in patients with T4 rectal cancer or fixed T3 tumors, compared to standard treatment, does not result in differences in DFS, OS, local recurrences, or distant metastases over an 8-year period [177].

A meta-analysis demonstrates a better 3-year DFS following S-RT plus CNCT (RR, 1.08; 95% CI, 1.01–1.14) or INCT plus LCRT (RR, 1.12; 95% CI, 1.01–1.24) compared to standard therapy, despite the increased 5-year LR rate for S-RT plus CNCT (RR, 1.65; 95% CI, 1.03–2.63) [173].

Remarkable results were presented by another study [99], which showed that TNT significantly reduces distant metastases, increases DFS by 5–10%, and ultimately improves OS by approximately 5% at 7 years. Three-year survival after a NOM approach exceeded 40% or 50%, depending on whether patients received INCT or CNCT. Similar y5DFS rates were observed in patients treated with INCT and CNCT [106].

Another meta-analysis found that INCT was the best treatment for DFS and 3-year OS (SUCRA 0.72 and 0.87, respectively), as well as at 5 years [174]. Consequently, the optimal sequencing of TNT remains a debated topic and should be considered individually for each patient.

### 8.5. NOM Rate

Some authors have classified the clinical tumor response after TNT into cCR, near-complete response (nCR), and incomplete clinical response to maximize patient eligibility for NOM. Patients with an incomplete clinical response after TNT undergo surgical treatment, while those with cCR or nCR may be offered an NOM strategy. The probability of NOM at 3 years was 77% (95% CI, 70–85%) for patients with cCR and 40% (95% CI, 32–51%) for those with nCR (*p* < 0.001) [139].

Some studies have shown that TNT led to long-term organ preservation in half of the patients, while CNCT resulted in a higher 5-year organ preservation rate compared to INCT [131]. Several trials (RAPIDO, UNICANCER-PRODIGE 23, CAO/ARO/AIO-12, and STELLAR) indicate that the NOM rate is higher than pCR rates in patients treated with TNT and TME [36,37,58,104].

Other studies report that only 15% of patients with cCR achieve pCR [140,158]. These differences are likely associated with the use of TNT, the total duration of treatment, and tumor response evaluation approximately 8 weeks after the completion of TNT.

A meta-analysis found that patients with cCR who underwent surgery had significantly better outcomes than those who followed an NOM strategy in terms of LR rate, y3DFS, y5DFS, and y5OS. The authors recommended NOM only for patients with poor biological status and high surgical risk [30].

While TNT can improve pCR and DFS, definitive curative treatment still relies on complete surgical resection as the standard of care.

## 9. Surgical Management

Currently, rectal cancer surgery is highly specialized, particularly for performing TME and extended lateral pelvic lymph node dissections and involving a multidisciplinary team in cases of planned extensive resections [178].

### 9.1. Current Surgical Principles

For a substantial proportion of patients with locally advanced rectal cancer (LARC), surgery represents the second stage after neoadjuvant therapy, where transabdominal rectal resection is the treatment of choice. The removal of the primary tumor should be performed with clear resection margins (CRM) and appropriate distal resections. Practically, an extension of 4–5 cm below the distal tumor margin is recommended, or for distal tumors (<5 cm from the anal verge), a 1–2 cm free margin is acceptable. Total rectal mobilization is useful for performing TME. TME is a standard component of radical rectal surgery, alongside the removal of the lymphatic drainage area. Regarding lymph node dissection, suspicious adenopathies beyond the resection margins should be biopsied or removed if possible. Extensive resection of M1 adenopathies is not indicated but may be performed based on imaging or intraoperative findings of suspicious adenopathies [179,180]. Simultaneously, sphincter preservation and organ integrity must be maintained without compromising oncological resection, taking into account the functional outcome and the quality of life expected by the patient.

For patients where a restorative procedure is not possible due to tumor location, invasion, or involvement of the sphincter complex, which may affect continence, an abdominoperineal resection may be the best surgical option. In the absence of these characteristics, sphincter preservation with restorative proctectomy is usually feasible. In restorative proctectomy after neoadjuvant therapy, a defunctioning stoma is typically created to reduce the morbidity associated with anastomotic fistulas.

Current neoadjuvant therapies included in the TNT strategy offer distinct advantages for surgery by reducing tumor volume, downstaging, achieving a pCR, and enabling sphincter-preserving resections [36,37,58].

Minimal invasive surgery has proven to be safe [181,182], with no significant differences in DFS and LR rates when compared to open surgery [183,184]. However, minimally invasive surgery is not recommended for LARC cases with large tumor volume or CRM involvement, or in the case of obstruction or perforation, for which open surgery is preferred.

Surgery, as the cornerstone of multimodal treatment, has become the final stage in TNT protocols or may even be omitted from the protocol if organ-preserving strategies (NOM) are adopted. This evolution has been driven by the fact that rectal adenocarcinoma is a disease with a high recurrence rate, as well as high morbidity associated with the different types of treatments [185].

### 9.2. Postoperative Complications

Rectal cancer surgery is associated with numerous postoperative and long-term complications that affect the quality of life: anastomotic fistulas with an incidence of 10–30% and postoperative mortality of 2–5% [186], sexual dysfunctions, found in up to 90% of patients [187], urinary dysfunctions, common in up to 38% at 5 years [188], and low rectal resection syndrome (LARS), a complication specific to rectal surgery, affecting 50% of patients at 13 years [189]. A recent retrospective study demonstrates the presence of postoperative complications in patients with RC operated on by low anterior resection (LAR) and ultra-low anterior resection (ULAR) in 26.31% of cases, with 80% of them having received neoadjuvantchemoradiation therapy (LCRT) [190].

The TNT strategy is associated with a morbidity rate of approximately 50%, including 20% pelvic infections (abscesses and anastomotic fistulas), 10% obstructions, and 20% medical complications [186]. Approximately half of the patients experience functional sequelae such as digestive disturbances (diarrhea, constipation, anal incontinence) or genitourinary disorders (impotence, anejaculation, and urinary incontinence) [191]. The Canadian study highlights increased tissue friability and edema following TNT compared to conventional RT [163].

Regarding fistula complications, the literature presents contradictory results. The increased risk of developing anastomotic fistulas after neoadjuvant CRT is highlighted in retrospective studies [192,193]. The pathogenic mechanisms involved in the development of anastomotic fistulas after neoadjuvant RT are based on increased inflammatory response in the rectal wall and pelvic area [194], damage to connective tissue [195], and effects on the microvascularization of the intestinal wall and pelvic tissues, which result in vascular obstruction with reduced blood flow, leading to poor wound healing.

In a study analyzing different neoadjuvant treatment regimens, it was found that 20.2% of patients who underwent CRT developed an anastomotic fistula, 23.6% of those with a combined regimen including oxaliplatin, and only 8.5% of those treated with exclusive 5FU (*p* = 0.007) [196]. Other studies demonstrate the lack of influence of neoadjuvant CRT on the occurrence of anastomotic fistulas [197,198].

Although its etiology is multifactorial, there are certain surgical risk factors, such as double-stapled anastomosis and suboptimal colonic vascularization. In this context, single-stapled anastomosis appears to reduce the fistula rate [199]. Real-time angiography with indocyanine green to assess colonic blood supply seems to reduce fistula rates in rectal cancer surgery [200], although the results of the forthcoming European multicenter prospective IntAct RCT study are eagerly awaited [201].

Low rectal anastomotic resection syndrome (LARS) occurs after rectal resections with TME and sphincter preservation. It is defined as the presence of functional disorders of the colon following rectal resection that affect the quality of life, including fecal incontinence, urgency, stool clustering and fragmentation, and inability to defecate [16,202]. Its etiology is multifactorial, with the involvement of neoadjuvant CRT [203], anal sphincter lesions [204], low anastomosis location [205], the type of anastomosis [206], the presence of a diverting stoma, and the timing of its closure [207]. Neoadjuvant RT leads to reduced tissue elasticity [208], which induces LARS [205].

In a multivariate analysis conducted by Qiu et al., along with the low location of the anastomosis, preoperative RT and short postoperative recovery were significant risk factors for LARS [209]. Data from the literature on the relationship between the laparoscopic approach and LARS occurrence are limited. A retrospective study of laparoscopic surgery after neoadjuvant CRT demonstrated a high frequency of LARS, which was described in 35.9% of patients, with the risk factors being neoadjuvant treatment and low tumor location [210].

Given its multifactorial etiopathogenesis, LARS management is complex, requiring time and a multidisciplinary team approach, as it significantly impacts the patient’s quality of life.

Neoadjuvant RT is a determinant factor in the occurrence of surgical site infection (SSI). The Stockholm study indicates an increased risk of SSI in patients receiving RT compared to those undergoing surgery alone (14% vs. 9%) [211]. Furthermore, CRT plays an important role in the occurrence of SSI after rectal cancer surgery, being present in 18% of patients treated with CRT and only 11% of those operated on primarily [212]. S-RT not only increases the incidence of wound infections but also represents a risk factor for perineal infections and pelvic abscesses after rectal amputation [51]. Another study shows that CRT is closely associated with wound and perineal infections. Possible causes include impaired microcirculation, with obstructive vasculitis and delayed healing [213].

The time interval until surgical intervention following TNT remains a topic of debate among surgeons, as pelvic fibrosis can make surgery more difficult and consequently increase postoperative morbidity.

## 10. Non-Operative Management (NOM)

Considering that patients with LARC who undergo neoadjuvant therapy and surgical interventions face various quality-of-life issues, including the need for colostomy, urinary problems, sexual dysfunction, and 50% of them experiencing LARS, this type of strategy seems particularly attractive, especially for the patient.

The WW strategy is considered to improve the quality of life for patients with cCR, multiple comorbidities, or those who reject surgery, without compromising survival.

In this regard, studies such as the updated 2023 OPRA study [106] demonstrated that the goal of organ preservation to avoid the morbidity of TME is achieved through TNT and the WW strategy in half of the patients. TNT in the sequence of CNCT is more effective than INCT. Salvage surgery in patients who developed tumor regrowth during the WW strategy had a similar 5-year DFS compared to those who underwent surgery for incomplete clinical response. The 5-year local recurrence-free survival rate was 94% vs. 90%, and the 5-year OS was similar between the two groups.

These results suggest that the WW strategy is safe for patients with cCR or nCR after TNT. Despite these encouraging results, further research through prospective multicenter studies is required to confirm the non-inferiority of this strategy. Currently, TNT has been incorporated as part of treatment algorithms in the most recent guidelines (NCCN 2024), but only in specialized centers with multidisciplinary experience for patients with excellent compliance withthe intensive follow-up program [105].

Studies such as TORCH [214] or NOMINATE [215] will prospectively evaluate quality-of-life differences between patients undergoing surgery or WW after complete response in patients treated with TNT.

The main concern is the potential for distant metastasis in patients during the follow-up period. Although this can be addressed through radical surgery or systemic therapy, there remains a life-threatening possibility in 5–15% of cases [159].

Implementing this strategy as a standard practice in older patients or those with less advanced distal tumors, where avoiding abdominoperineal resection is paramount, is a clinical challenge that must be carefully evaluated.

## 11. Perspectives

Several important directions are emerging for future research. One would be the identification of appropriate patient selection criteria for different strategies (CRT, TNT, and CT only) based on predictive factors for treatment response, the identification of therapeutic strategies with maximum efficacy, and the development of an optimal method for assessing the response to neoadjuvant treatment, particularly in terms of the risk of LR or systemic relapse in the case of the WW strategy.

The purpose of proper preoperative staging is to facilitate treatment planning by identifying patients who are most likely to benefit from neoadjuvant therapy and to assist in selecting the optimal surgical procedure. Prediction models could be further improved by adding new biomarkers, such as circulating tumor biomarkers, molecular profiling, or pathological immune biomarkers. While the predictive value of some markers, such as tumor stage, has been well characterized, others, such as the tumor immune microenvironment and the intestinal microbiome, are still under development.

One study concluded that high-quality MRI can select patients with a low risk of LR who are likely to have good outcomes without RT. The high-risk group, which benefits from neoadjuvant RT, presents MRI-proven prognostic factors (EMIV, tumor deposits, and CRM+) [216].

Recently, genetic prediction models for neoadjuvant treatment response have been developed. The PGS-LARC model [217] includes 15 genetic variants and has a positive predictive value for complete response of 94.7%. Some of the genes included are involved in oxidative stress (SIPA1L2, DPP3, HORMAD1, and MRPL18), while others are involved in immune response (HLA-DPB1, CD82, and DIP2A). This model, in combination with cCR, accurately identified patients who would achieve a pCR, providing strong support for the WW strategy.

Some authors propose a model based on patient-derived organoids (PDO) to identify patients with LARC who do not respond to neoadjuvant CRT, selecting them for further investigations regarding targeted therapy. PDO-based treatment response tests take about 4 weeks, allowing timely treatment recommendations [218].

Another research direction addresses the intestinal microbiome. The NCT05079503 study investigates the impact of the intestinal microbiota on treatment response in rectal cancer. The authors suggest that the probiotic GEN-001 (Lactobacillus lactis) may activate CD4+ or CD8+ T cells and NK cells, exhibiting synergistic effects with oxaliplatin-based CT [219].

However, few predictive markers are used in clinical routine, as their potential utility is limited due to the heterogeneity of biomarker studies, lack of validation on independent cohorts, or the absence of a marker panel with substantial predictive power. To address these shortcomings, future clinical studies should be complemented by biomarker discovery programs, which include the collection of tumor tissues, blood, and stool samples for comprehensive multi-omics analysis. In the future, predictive biomarkers will likely become an essential part of managing patients with LARC.

Since TNT leads to pCR rates of nearly 30%, the WW strategy has been increasingly debated for responders. The critical point is the correct identification of potential candidates for the WW strategy without compromising oncological safety.

Response evaluation has become a key point, with clinical and radiological assessments having limited efficiency. Promising tools that integrate radiomic analysis of pre- and post-treatment MRI, PET-CT, and CT scans, along with clinicopathological data and molecular biomarkers within machine learning algorithms, have the potential to accurately predict treatment response and asses the risk of local or systemic recurrence.

## 12. Conclusions

The adoption of the TNT strategy in patients with LARC is recommended for those with a high risk of LR or distant metastases. Identifying patients with LARC for TNT requires thorough MRI imaging analysis, various tumor genetic tests, and a multidisciplinary team analysis for the selection and determination of the appropriate type of TNT. For patients with cCR, the continuation of treatment remains a challenge due to the lack of precise means forevaluating the risk of LR or systemic relapse within the context of the WW strategy.

Ongoing clinical studies continue to focus on the development of the most effective treatment for LARC while also considering the impact on the patients’ quality of life. On the other hand, it is essential to apply a personalized treatment plan adapted to the patient’s options, based on a thorough diagnosis of the tumor.

## Data Availability

Not applicable.

## Supplementary Materials

The following supporting information can be downloaded at: https://www.mdpi.com/article/10.3390/life15071011/s1, Table S1: Advanced search in PubMed data base for RC and therapy/neoadjuvant therapy/TNT.

## Abbreviations

The following abbreviations are used in this manuscript:

RC	rectal cancer
MRF	mesorectal fascia
CRM	circumferential resection margins
CRT	chemoradiotherapy
CT	chemotherapy
LCRT	long-course radiotherapy
S-RT	short-course radiotherapy
pCR	pathologic complete response
cCR	clinic complete response
NOM	non-operative management
WW	wait-and-watch strategy
