# Supplementary material for: Controversies and Perspectives in the Current Management of Patients with Locally Advanced Rectal Cancer—A Systematic Review
Source: Life (Basel). 2025 Jun 25;15(7):1011. doi: 10.3390/life15071011 (PMC12299300; doi:10.3390/life15071011)
Supplement: Supplementary file 1 [file life-15-01011-s001.zip › life-3644365-supplementary.pdf]

**Table S1** Advanced search in PubMed data base for RC and therapy/neoadjuvant therapy/TNT

| Search | Query | Results |
|---|---|---|
| #5 | Search: **((rectal cancer) OR (rectal neoplasia)) AND (total neoadjuvant therapy)** Filters: **Full text, Clinical Trial, Meta-Analysis, Observational Study, Randomized Controlled Trial, Review, Systematic Review, English, Humans, Adult: 19+ years, from 2015 - 2025** | 234 |
| #4 | Search: **((rectal cancer) OR (rectal neoplasia)) AND (total neoadjuvant therapy)** Filters: **Full text, Clinical Trial, Meta-Analysis, Observational Study, Randomized Controlled Trial, Review, Systematic Review, English, Humans, from 2015 - 2025** | 556 |
| #3 | Search: **((rectal cancer) OR (rectal neoplasia)) AND (total neoadjuvant therapy)** Filters: **Full text, Clinical Trial, Meta-Analysis, Observational Study, Randomized Controlled Trial, Review, Systematic Review, English, Humans** | 784 |
| #2 | Search: **((rectal cancer) OR (rectal neoplasia)) AND (neoadjuvant therapy)** Filters: **Full text, Clinical Trial, Meta-Analysis, Observational Study, Randomized Controlled Trial, Review, Systematic Review, English, Humans** | 1,771 |
| #1 | Search: **((rectal cancer) OR (rectal neoplasia)) AND (therapy)** Filters: **Full text, Clinical Trial, Meta-Analysis, Observational Study, Randomized Controlled Trial, Review, Systematic Review, English, Humans** | 9,178 |

Final number after review for inclusion and exclusion criteria and addition of articles from review of references[a]

[a]Exclusion criteria: No abstract available; non rectal cancer; TNT not a major focus of article; case reports without TNT;
